# MR scan evaluation of pelvic organ prolapse mesh complications and agreement with intra-operative findings

**DOI:** 10.1007/s00192-019-04182-7

**Published:** 2019-12-18

**Authors:** Charlotte Mahoney, Adam Hindle, Balashanmugam Rajashanker, Rohna Kearney

**Affiliations:** 1 The Warrell Unit, St Mary’s Hospital, Manchester University Hospitals NHS Foundation Trust, Manchester Academic Health Science Centre, Oxford Road, Manchester, M13 9WL UK; 2 Fairfield General Hospital, Pennine Acute NHS Trust, Bury, BL9 7TD UK; 3 Department of Radiology, Manchester Royal Infirmary, Manchester University Hospitals NHS Foundation Trust, Manchester Academic Health Science Centre, Manchester, M13 9WL UK; 4 Institute of Human Development, Faculty of Medical & Human Sciences, University of Manchester, Manchester, UK

**Keywords:** MR scan, Sacrocolpopexy, Sacrohysteropexy, Mesh complications

## Abstract

**Introduction:**

An increasing number of women are presenting with symptoms after the placement of mesh implants for prolapse which may be attributable to a mesh implant complication. MRI imaging can be used to evaluate abdominally placed mesh but there is no published research evaluating the use of MRI in this group of women. The objective of our study was to report our experience as a tertiary centre in evaluating abdominal mesh with MR imaging and the agreement of MR reports with surgical findings.

**Study design:**

A retrospective observational cohort study (Canadian Task Force classification II-2) of all women referred to our tertiary unit who underwent an MR scan for investigation of symptoms of mesh complication following an abdominally placed mesh implant between June 2006 and September 2018 was performed. The reports of MR images were compared with the findings at surgery.

**Results:**

MR scan was performed in 87 with suspected mesh complications. MR scan detected mesh failure in 42.1% of women (37/87), infection in 12.6% (11/87), compression in 2.3% (2/87), exposure in 12.6% (11/88), bowel extrusion in 2.3% (2/87) and inflammation in 11.5% (10/87). Agreement between MR scan report and surgical diagnosis was almost perfect for mesh failure, infection and compression, whilst agreement was only moderate for mesh erosion and signs of inflammation (failure κ = 0.97, infection κ = 0.94, compression κ = 1.0, exposure κ = 0.58 and inflammation κ = 0.24).

**Conclusion:**

These data provide information on the role of MR imaging in the investigation of women presenting with suspected intra-abdominal POP mesh complications including recurrence.

## Introduction

One in three women will experience pelvic organ prolapse (POP) within their lifetime, and between 4 to 12% of these will involve the apical compartment, either the uterus or the vaginal vault post-hysterectomy [1]. Sacrocolpopexy and sacrohysteropexy surgical procedures use synthetic mesh to suspend the uterus or the vaginal vault to the sacrum and can be performed by an open, laparoscopic or robotic approach. Although published success rates for these procedures are good, recognized complications include mesh exposure and extrusion, pain, dyspareunia, mesh infection, fistula formation and mesh failure with recurrence of POP [2–4]. Whilst the long-term complication rate for abdominal POP mesh is uncertain, the VIGI-MESH registry reported short-term complication rates of up to 1.0% (0.1–1.9%) for laparoscopic sacropexy and rectopexy with mesh [5].

In recent years there has been an increase in the number of women presenting to our service with concerns and symptoms following mesh procedures for incontinence and prolapse. Our multidisciplinary team has used MR to evaluate women presenting with complications after abdominally placed mesh for > 10 years and anecdotally found this was helpful for pre-operative evaluation. There are no published data other than pictorial reviews of MR imaging in synthetic mesh implants.

The objective of this study was to retrospectively evaluate the MR reports and analyse their agreement with surgical findings.

## Materials and methods

### Patients and procedures

A retrospective single-centre cohort study was performed to describe the MR findings in women presenting with recurrence or suspected complications of abdominally inserted mesh for POP and compare this with the intra-operative findings. All women who present with symptoms that may be associated with a mesh complication after placement of abdominal mesh are evaluated by an MRI scan in our unit. Evaluation of mid-urethral slings and vaginally inserted POP mesh were excluded as women are offered translabial ultrasound to evaluate these mesh implants in our unit.

Mesh failure was defined as a recurrence of POP in the apical compartment. Mesh findings were grouped into mechanical failure, infection, compression, exposure and extrusion and inflammation.

Women with POP recurrence and/or suspected mesh complication following an abdominal mesh procedure performed at any hospital, who presented to our tertiary urogynaecology unit between June 2006 and September 2018 and underwent an MR scan, were included in the study. Women were excluded if they did not have an MR scan. The unit was registered with the British Society of Urogynaecology as a mesh complication centre in July 2017.

### Ethical approval

The UK health research authority which governs all research in the UK designates a study of this nature as a service evaluation that does not require ethical approval; however the study protocol was performed in compliance with the Declaration of Helsinki [6].

### MRI imaging protocol

MR scans were performed using 1.5- and 3-T MR scanners, high-resolution axial, coronal and sagittal T2W, axial T1W and axial T2 fat saturated (3-mm slice thickness, FOV 26–30, matrix size 256 × 192), at our hospital or at the referring hospital. Scans were repeated at our hospital if after review the sequences obtained were insufficient for evaluation of the mesh.

All MR scan images were initially reviewed by one of three consultant radiologists specializing in genitourinary radiology who have been working as part of the multidisciplinary pelvic floor team. Over a 10-year period they have developed skills in evaluating polypropylene mesh used in the treatment of pelvic organ prolapse and urinary incontinence. A second review occurred at a multidisciplinary meeting between radiology and urogynaecology, including the surgeon.

### Study protocol

Women undergoing MR imaging were identified from the urogynaecology-radiology multidisciplinary team meeting and a previous audit of mesh complications. Paper case notes and electronic data systems were hand-searched for information regarding age, presenting complaints, examination findings in the clinic, location of previous mesh implant(s), MR scan and intra-operative findings. Operations were performed at the tertiary unit by one of three subspecialist surgeons trained in laparoscopic urogynaecology. All surgical procedures were recorded on DVD allowing further verification of operative findings.

MR scan reports were viewed and compared with the corresponding operation notes and DVDs of the surgical procedures.

All data were collected by one researcher and cross checked by a second researcher for standardization.

### Statistical analysis

Statistical analysis performed using Stata version 15.0 (StataCorp, College Statin, TX, USA) and mesh complication categories defined a priori in the plan of analysis. Diagnosis under direct vision at surgery was considered the gold standard and agreement with MRI scan was compared using Cohen’s kappa (poor < 0.0, slight = 0.00–0.20, fair = 0.21–0.40, moderate = 0.41–0.60, substantial 0.61–0.80, almost perfect 0.81–1.00) [7].

## Results

During the study time frame 87 women had an MR scan following abdominal insertion of mesh for pelvic organ prolapse (POP). Fifty-eight women went on to have surgery on the apical compartment and a further five women were awaiting surgery at the time of writing. Of the remaining 24 women, 7 opted for conservative management, 6 were referred to another specialty (upper gastrointestinal, colorectal, orthopaedics and pain), 4 declined further surgery, 4 were discharged after a reassuring normal MR scan, 2 were considering their treatment options and 1 opted for referral to a private centre.

Seventy-six women attended with more than one presenting complaint, the most common being recurrence of POP and the next was pain (Table 1). Again, the most common finding on examination was a recurrence of POP and the next was provoked pain on vaginal palpation of the mesh. The most common mesh under evaluation was following a sacrocolpopexy (Table 1).

**Table 1:** Cohort characteristics and mesh implants

*Age, years* Median (range)			56 (30–83)
*Time from surgery to presentation, years* Median (range)			2, (0.5–14)
*Presenting complaint* *Frequency (%)*	Symptoms of POP Pain Urinary dysfunction Bowel dysfunction Discharge Dyspareunia Hispareunia		52 (59.8) 35 (42.5) 32 (36.7) 19 (21.8) 17 (19.5) 7 (8.0) 1 (1.1)
*Examination findings* *Frequency (%)*	POP Provoked pain Discharge Exposure		54 (62.1) 17 (19.5) 12 (13.8) 16 (18.4)
Mesh implants			
*Abdominally implanted apical prolapse mesh* *Frequency (%)*	Sacrohysteropexy Single sacrohysteropexy More than one sacrohysteropexy Sacrocolpopexy Single sacrocolpopexy More than one sacrocolpopexy		33 (37.9) 31 2 54 (62.1) 51 3
*Additional mesh implant(s)* in situ *Frequency (%)*	*Vaginal*	Anterior Posterior Vault	4 3 1
	*Mid-urethral sling*	TVT TOT Other	9 1 1
	*Intraabdominal*	Rectopexy	7

Key: POP, pelvic organ prolapse; TVT, transvaginal tape; TOT, transobturator tape

Note: 56 women attended with more than one presenting complaint. Twelve women had more than one examination finding. Sixty-four women had received a single mesh implant, 17 women had received 2 implants, 4 women had received 3 implants and 2 women had received 4 implants

Twenty-six women had more than one prior mesh placement of a vaginal mesh, mid-urethral sling or rectopexy. In these cases the history and examination findings were correlated with the location of all previous mesh implants to distinguish the abdominal prolapse mesh as the cause of their symptoms.

### Diagnosis

Thirty-three MR scans (37.9%) were performed to evaluate sacrohysteropexy mesh and 54 (62.1%) to evaluate sacrocolpopexy mesh. Twenty-three women had a normal MRI scan. The sacrocolpopexy or sacrohysteropexy mesh was identified by the radiologist in all cases.

#### Mesh mechanical failure

**Fig. 1:**
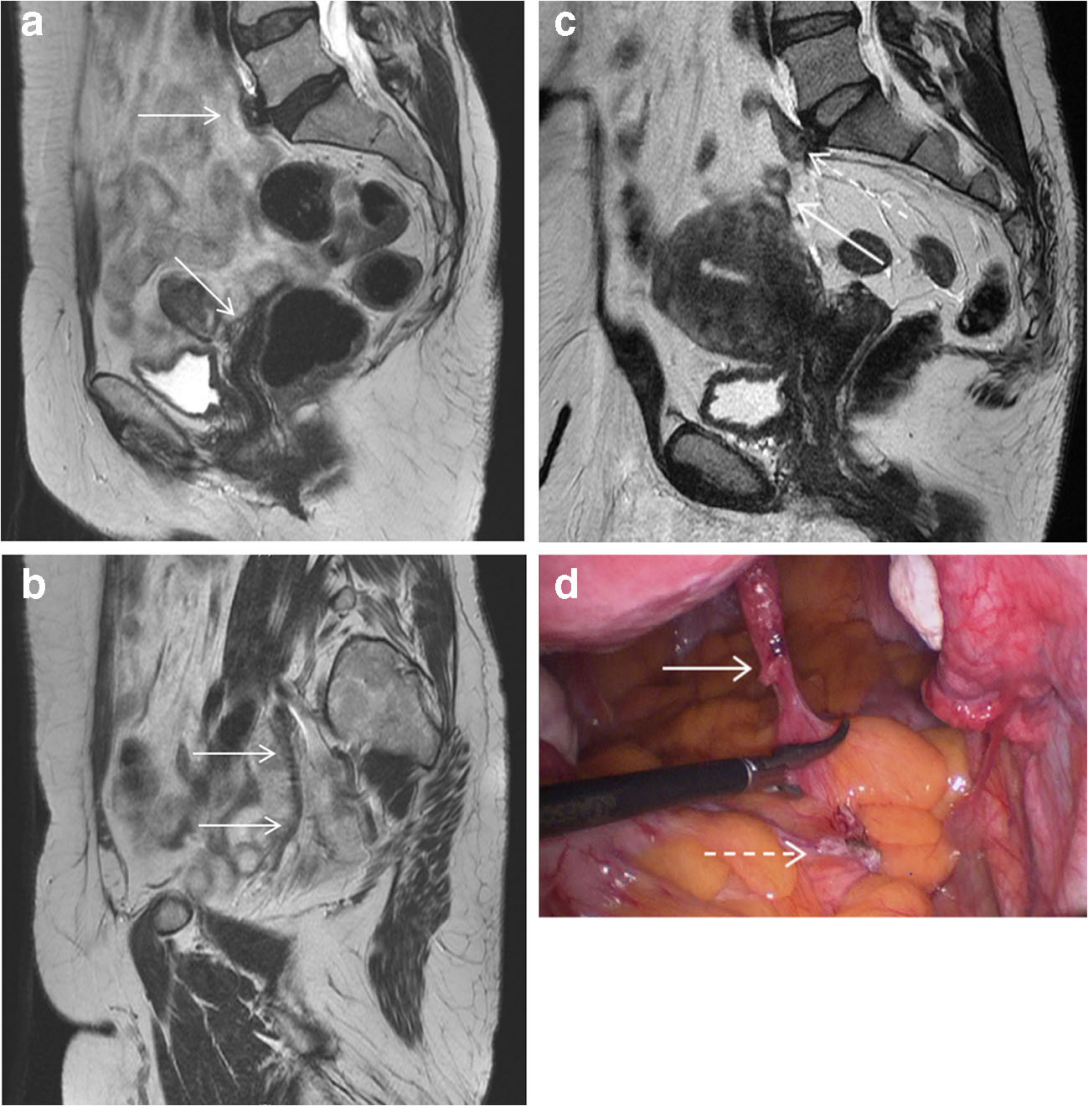
MR scan (**A**, **B** and **C**) and a laparoscopic image (**D**) showing mechanical mesh failure. **A** and **B** Mesh laxity on MR scan. In **A**, taken from the midline, the sacral and vaginal attachments of the mesh can be seen (arrows) but the mesh cannot be traced through the pelvis. In **B**, taken from close to the right pelvic sidewall, the mesh can be traced around the edge of the pelvis (arrows). The mesh is lax as it follows an indirect route along the pelvic sidewall from the sacrum to the vagina instead of a more direct course through the pelvis. **C** and **D** show detachment of the mesh from the sacral promontory. C shows the free end of the mesh within the abdominal cavity on MR scan (solid arrow) and a small remnant of mesh at the sacral promontory (dashed arrow). The mesh can be traced from the sacral and vaginal attachments but the two halves of the mesh do not meet, indicating there is a break in the mesh. **D** is the corresponding laparoscopic image showing the free end of mesh lying within the abdominal cavity (solid arrow) separate from the small remnant of mesh on the promontory (dashed arrow)

Mechanical mesh failure was defined as mesh laxity whereby the mesh follows an indirect route along the pelvic sidewall from the sacrum to the vagina or cervix instead of a direct course through the pelvis, breakage or detachment from the vagina, cervix or sacral promontory. MR scan detected mesh failure in 37 women (42.1%) (Fig. 1). Two women were diagnosed with complete mesh detachment from both the sacral promontory and vagina. Of the 39 cases of mesh failure 13 (33.3%) had mesh laxity, 12 (30.8%) detachment from the sacral promontory, 12 (30.8%) vaginal or cervical detachment and 2 (5.1%) mesh breakage.

Of the 58 women who opted for further surgery, mesh failure was reported in 25 women (42.4%) on MR scan and diagnosed in 26 women (44.1%) at laparoscopy (laxity 40% vs. 42.3%, sacral detachment 20% vs. 23.1%, vaginal or cervical detachment 40% vs. 38.5%, and mesh breakage 8.0% vs. 7.7% respectively).

There were no cases of mesh failure reported on MR scan that were not corroborated on surgical diagnosis.

One case of mesh failure was not reported on MR scan but was diagnosed intra-operatively following presentation with deep vaginal pain. The MR scan reported extensive bowel adhesions around the vaginal portion of the mesh with extrusion of mesh into the small bowel. The woman underwent laparoscopy, which was converted to laparotomy, division of bowel adhesions, small bowel resection including the area of mesh extrusion, excision of vaginal mesh exposure and removal of entire sacrocolpopexy mesh. During this extensive surgery it was noted the mesh ended blindly within the mesentery and was not attached to the sacrum.

#### Infection

Mesh was classified as infected if there were signs of a loculated collection, tracking abscess, sinus tract or osteomyelitis. MR scan found evidence of infection in 11 women (12.6%); 2 women were diagnosed with a loculated collection that had extended to the level of the sacrum. Of these 13 cases, a loculated collection was present in 9 (69.2%), a sinus tract in 2 (15.3%) and the infection extended to the level of the sacrum in 2 cases (15.3%).

One woman was initially diagnosed with a granulomatous mass associated with infected mesh; however following upper gastrointestinal radiology and MDT oncology review she was found to have an abdominal recurrence of pancreatic cancer.

**Fig. 2:**
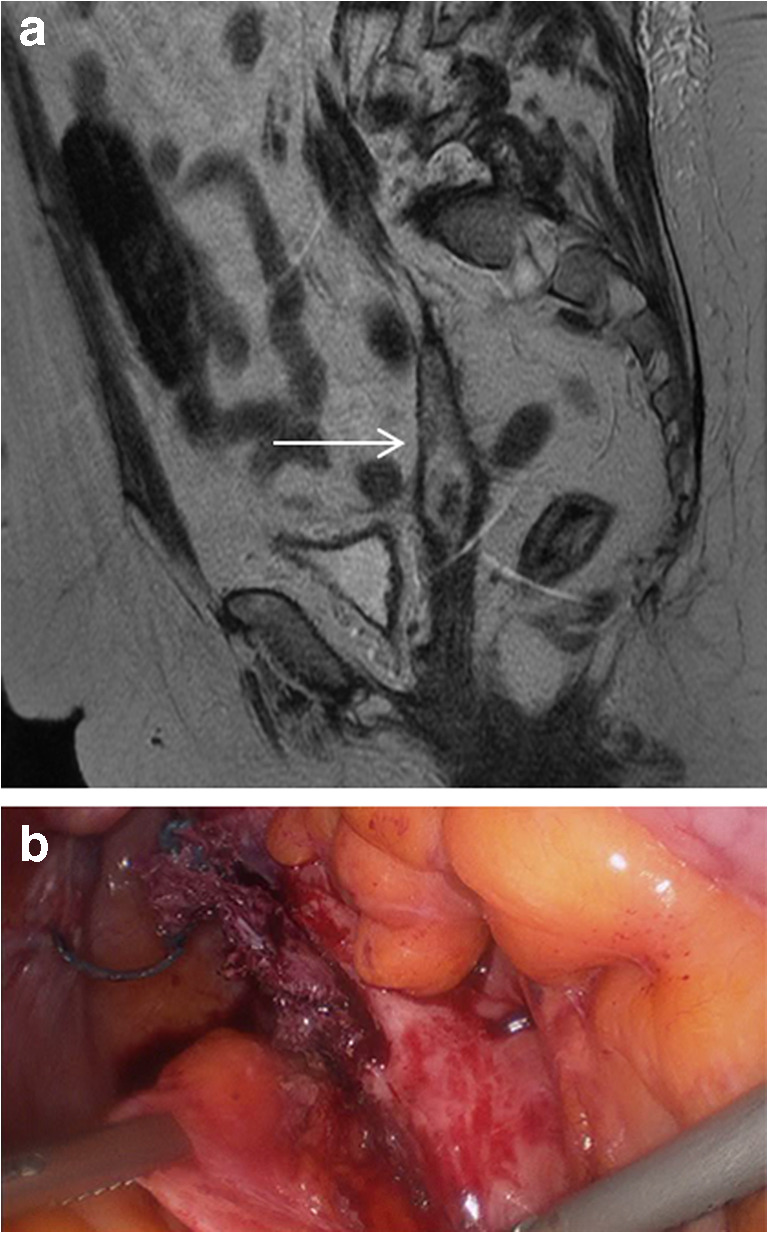
MR scan (**A**) and corresponding laparoscopic image (**B**) of an infected mesh tract. **A** MRI demonstrating an infected mesh tract with vaginal wall erosion (solid arrow) and the corresponding laparoscopic image (**B**) from the same woman demonstrating the partially excised thickened infected mesh tract

Of the remaining ten women who underwent laparoscopy, all were found to have evidence of mesh infection intra-operatively. A sinus tract was present in seven women (70%), a loculated collection in three women (30%) and infection extending to the level of the sacrum in two women (20%). See Fig. 2.

#### Mesh compression

**Fig. 3:**
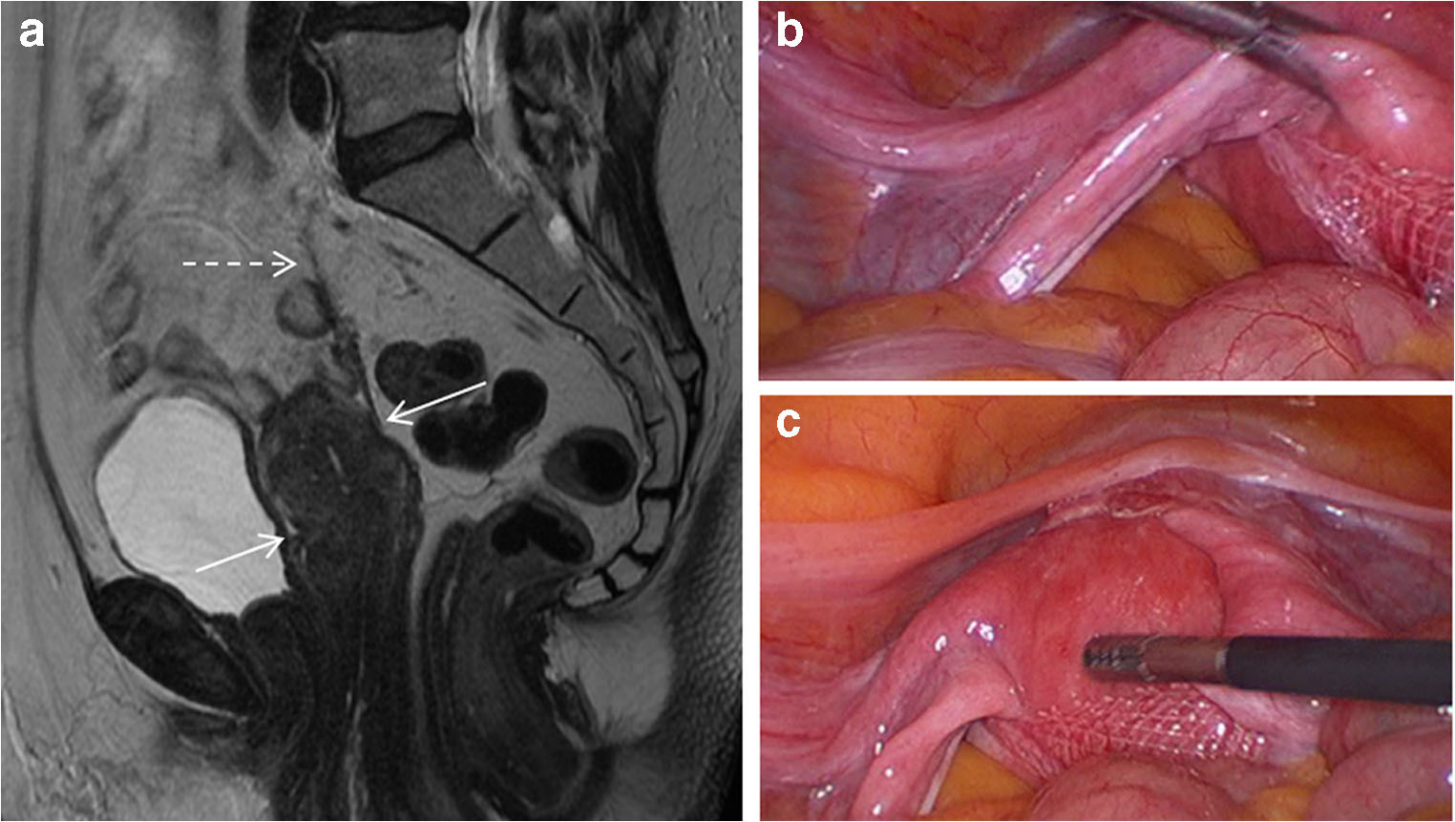
MR scan (**A**) and corresponding laparoscopic images (**B** and **C**) of uterine compression by sacrohysteropexy mesh. **A** Sagittal MRI showing compression of the uterus (two solid arrows) with uterine prolapse with intact but slightly lax mesh (dashed arrow). **B** and **C** Corresponding laparoscopic images for the same patient which shows the uterus prolapsed through the arms of the sacrohysteropexy mesh

In two women (2.3%) the mesh was reported as being compressed; when this was discussed at the multidisciplinary team meeting the radiologist reported that in one case the uterus was seen as being compressed by the mesh, and in the other case the mesh was compressed between the uterus and a right ovarian cyst. At laparoscopic assessment in the first case the uterine fundus was found to have dropped through the sling created by mesh attachment to the anterior cervix through the broad ligaments (Fig. 3). In the second case a 7-cm ovarian cyst was found at surgery. Both women had presented with lower abdominal pain and discomfort.

#### Exposure and extrusion

Exposure was diagnosed in 11 women (12.6%) on MRI scan, with vaginal exposure reported in 10 women and cervical exposure in 1. In comparison mesh exposure was diagnosed on direct vision during outpatient clinical examination and intra-operatively in 19 women (21.8%) consisting of 13 cases of vaginal exposure and 6 cases of cervical exposure.

**Fig. 4:**
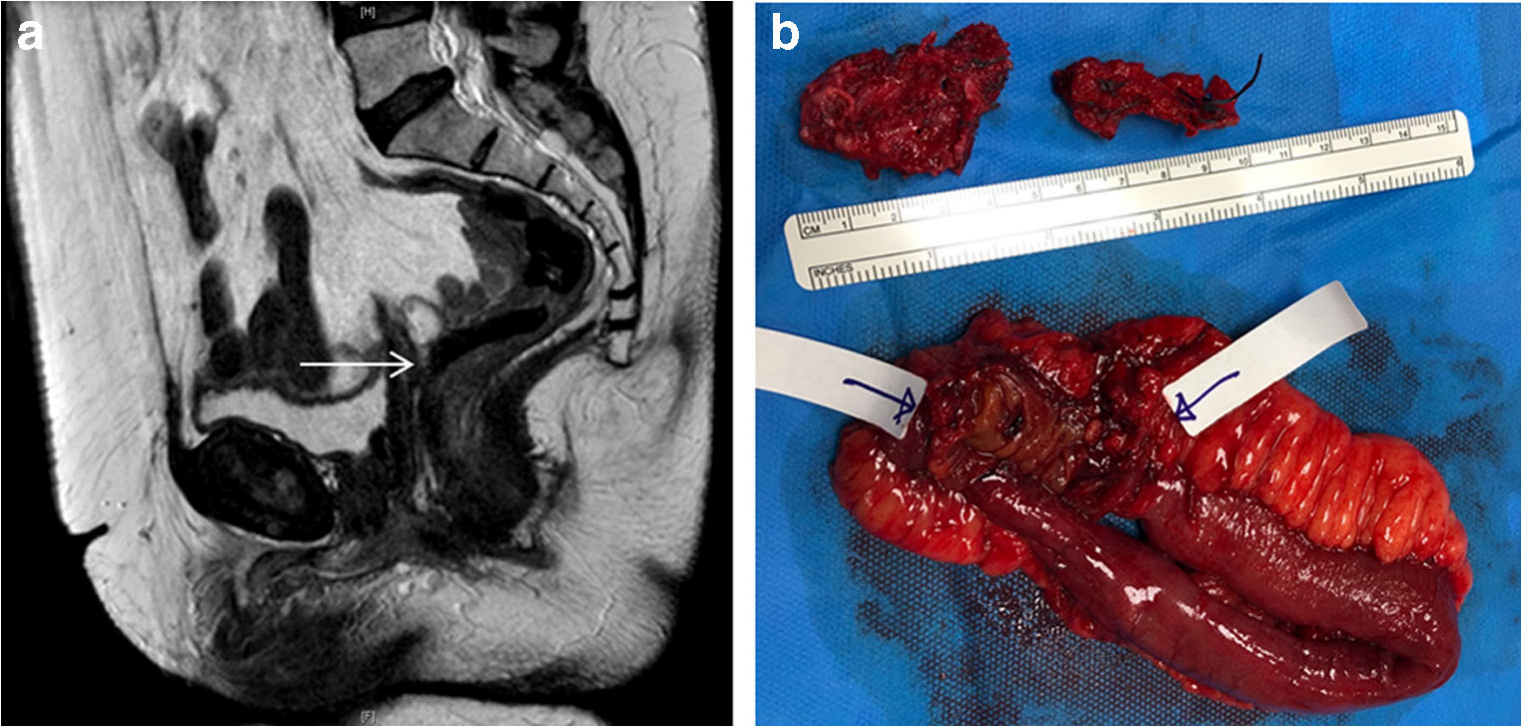
MR scan (**A**) and corresponding small bowel with mesh extrusion (**B**). **A** Bowel adhesions around the vaginal portion of the mesh with vaginal exposure and probable bowel extrusion on MR scan (solid arrow). **B** The portion of small bowel that was resected and the two pieces of mesh involved in the bowel extrusion

Bowel extrusion was reported in two women (2.3%) on MR scan. One case of reported bowel extrusion was not present at the time of examination under anaesthetic; instead a vesico-vaginal fistula secondary to a squamous cell carcinoma of the vagina was found. The second case was the woman described in the section on mesh failure who underwent a small bowel resection and complete mesh excision with resolution of her symptoms (Fig. 4).

#### Signs of inflammation

Adhesions, granulation tissue and thickening of tissues were reported in ten women (11.5%) on MR scan. Adhesions were reported in five women, granulation tissue in one woman, adhesions and granulation tissue in one woman and thickening of tissues in three women. These signs of inflammation were reported in cases of infected mesh, mesh failure, women presenting with provoked pain and dyspareunia.

Eight women went on to have surgery. Adhesions were present in three cases, granulation tissue in one case and thickened tissues in one case.

### Diagnostic accuracy

Fifty-eight women went on to have surgery which allowed direct comparison between their MR scan report and intra-operative diagnosis under direct vision.

Agreement between MR report and intra-operative diagnosis under direct vision was almost perfect for the diagnosis of mesh failure (κ = 0.97, CI 0.71 to 1.0, *p < 0.001*), mesh infection (κ = 0.94, CI 0.69 to 1.0, *p < 0.001*) and mesh compression (κ = 1.0, CI 0.74 to 1.0, *p < 0.001*). Agreement was moderate for mesh exposure and extrusion (κ = 0.58, CI 0.35 to 0.81, *p < 0.001*) and fair for signs of inflammation (κ = 0.24, CI 0 to 0.50, *p = 0.032*) [7].

## Discussion

This is the first study to report data on the use of MR imaging to diagnose mesh complications in women following intra-abdominal apical compartment POP surgery as well as an evaluation of agreement with surgical diagnosis. We found MR scan for mesh complications can provide important information to help plan further management – whether the woman is seeking reassurance, wishes conservative management or further surgery. In addition, we found excellent agreement between the MR scan and intra-operative diagnosis for mesh failure, compression and infection.

To date, there are no published data on the use of MR scan to evaluate mesh complications following sacrocolpopexy or sacrohysteropexy although MR scan is recommended as part of an individualized assessment of mesh complications in the 2019 NICE clinical guideline [8]. The imaging protocol has been described in three review articles [9–11].

One study has reported MR imaging of the lumbosacral region pre-operatively and 1 year post-operatively in 30 women following laparoscopic sacrocolpopexy or vaginal hysterectomy with concomitant laparoscopic sacrocolpopexy. They found mesh insertion was not associated with bone marrow or soft tissue change on MR scan, suggesting any post-operative soft tissue or bone marrow changes should raise suspicion of an inflammatory process, such as mesh infection or osteomyelitis [12].

Two studies have reported data on the use of MR scan following incisional hernia repair. Both studies evaluated a polypropylene mesh similar to the meshes used in this study as well as polytetrafluoroethylene or Gore-Tex® mesh. In the first study the authors were unable to identify the polypropylene mesh in any patients on MR scan, whilst the Gore-Tex mesh was visible in all cases [13]. The second study reported similar findings, with the polypropylene mesh visible in only 23.1% of cases [14].

Polypropylene mesh within the pelvic cavity is more easily visualized compared with abdominal wall mesh. This is probably related to the similar signal of the mesh and anterior abdominal wall musculature. It is possible that better visibility in the pelvis is due to the surrounding pelvic fat tissue, which is bright on T2W images, compared with the mesh, which has very low T2 signal. It is also likely that the accuracy of detecting the mesh is increased by radiologists experienced in reviewing these images.

A number of studies have reported dynamic MR scan to objectively measure surgical outcomes following pelvic organ prolapse surgery.

One study performed dynamic MR scans in ten women after sacrocolpopexy. They described difficulty identifying the mesh in 30% of cases and one case of vaginal exposure that was associated with evidence of soft tissue reaction on MR scan [15]. However the study was limited by very small numbers. The mesh was identified in all MR scans in our study.

A series of German studies measured dynamic MR scan, clinical examination and a quality of life questionnaire after vaginal wall mesh repair pre-operatively and 12 weeks post-operatively in 80 women, 1 year in 69 women and 5 years in 26 women. They found dynamic MR scan was useful in evaluating both recurrence and de novo POP in a different compartment, but tended to overestimate recurrence compared to clinical examination [16–19]. The series did not include an evaluation of mesh visibility on MR scan.

Recent research has described the use of MR visible mesh to facilitate imaging in the event of future complications [20–23]. Whilst the initial data appear promising the long-term safety of these devices is unclear and further evaluation is needed before introduction into clinical practice. The role of such meshes in Urogynaecology remains uncertain given abdominally placed POP mesh can be identified on MR scan.

There are no studies describing the role of ultrasound to identify or evaluate abdominally placed POP mesh.

The median duration from index surgery to presentation in our cohort was 2 years with a range of 0.5 to 14 years. Our data suggest mesh complications should be considered in all women presenting with symptoms regardless of duration since insertion. One prospective study reported a 1.0% incidence of complications following abdominally placed mesh. This study reported combined intra- and post-operative complication rates over 12 months and included women undergoing mesh rectopexy [5]. Our study does not report the incidence of complications following abdominal mesh procedures but rather our experience of using MR imaging to evaluate symptoms. Many of the women included in this study were referred from other units. These differences may explain the variation in complications described in our study. Our study provides important data supporting the use of MR scan to investigate women with potential mesh failure or mesh complications following intraabdominal or vaginal POP surgery.

### Limitations

This was a single site, retrospective study which introduces the risk of selection bias. The risk was reduced by the fact all women with potential complications for intra-abdominal POP mesh were offered an MRI scan during this time frame and we have reported findings of these and at the time of surgery when surgery took place.

Unfortunately there is no current mesh implant registry in the UK and women are often not aware of the type or brand of mesh implanted. In addition, women were referred from primary, secondary and tertiary care, often independent of the initial implanting unit and so this information was unavailable. We attempted to mitigate against this by requesting a copy of the operation note from the implanting surgeon and when available the operation note informed the case discussion between the radiologist and urogynaecologist aiding interpretation of the MR scan. As an example, in some cases the radiologist noted the mesh appeared thickened but this was explained by the operation note record that two pieces of mesh had been placed. On other occasions the mesh was reported as inflamed but review of the surgical record showed that a thicker heavier weight mesh had been placed.

We have concluded from this study that correlation of mesh appearance on MR with previous surgical record is important in interpretation of the images. To aid clinical management the MR scans in our unit are reported by one of three consultant radiologists and reviewed in a multidisciplinary meeting with the urogynaecology team so that the interpretation of the scan can be informed by the clinical history and examination findings. In addition this process facilitates feedback of the surgical findings from earlier reported scans allowing for development of expertise.

### Interpretation

This study demonstrates MR scan can be a useful tool for investigating POP mesh failure and complications when reviewed by a radiologist experienced in mesh imaging. Reporting should be performed alongside the clinical history with input from the multidisciplinary team.

Our results suggest MR scan is a useful investigation in diagnosing mesh failure, infection, compression and extrusion. It is moderate at best for vaginal or cervical mesh erosion and poor for adhesions, granulation tissue or thickening of tissues. The gold-standard for diagnosing vaginal or cervical mesh erosion is examination in the out-patient clinic or as part of an examination under anaesthetic, and our findings would appear to support this. One possible explanation is epithelial surfaces of the vagina and cervix are more easily visualized on examination compared with imaging.

### Generalizability

All MRI scans were either performed or reviewed by one of three Consultant Radiologists specialized in mesh identification and evaluation. Identification of mesh on MRI scan can be technically challenging and in our experience requires a specific protocol with a non-portable MRI scanner and an experienced radiologist. Our findings may not be generalizable to smaller units that do not have the equipment or personnel for performing and interpreting these MRI scans.

### Overall

The findings from this study are important, particularly in the context of the increasing number of women presenting with mesh complications. Our data support the use of MR scan as a non-invasive technique that can be used to assess abdominal mesh complications and guide treatment as part of a multidisciplinary team approach in a designated mesh centre with input, where relevant, from urogynaecology, urology, colorectal and pain specialists. This allows a pre-operative discussion of the surgical treatment plan with the woman and surgical planning for the procedure.

Further research is needed with prospective studies as part of a national register to investigate this further. There is currently no standardized reporting system for intra-abdominal prolapse mesh on MR scan which can lead to inconsistent reporting and the development of a standardized reporting system also requires further research.
